# Narrow band imaging-assisted transurethral resection reduces the recurrence risk of non-muscle invasive bladder cancer: A systematic review and meta-analysis

**DOI:** 10.18632/oncotarget.13054

**Published:** 2016-11-03

**Authors:** Weiting Kang, Zilian Cui, Qianqian Chen, Dong Zhang, Haiyang Zhang, Xunbo Jin

**Affiliations:** ^1^ Minimally Invasive Urology Center, Shandong Provincial Hospital Affiliated to Shandong University, Jinan, Shandong, China; ^2^ Department of Emergency, Shandong Provincial Hospital Affiliated to Shandong University, Jinan, Shandong, China; ^3^ School of Medicine, Shandong University, Jinan, Shandong, China

**Keywords:** non-muscle invasive bladder cancer, narrow band imaging, white light imaging, resection, recurrence risk

## Abstract

**Context:**

Compared with white light imaging (WLI) cystoscopy, narrow band imaging (NBI) cystoscopy could increase the visualization and detection of bladder cancer (BC) at the time of transurethral resection (TUR). NBI cystoscopy could increase the detection of BC, but it remains unclear whether narrow band imaging-assisted transurethral resection (NBI-TUR) could reduce the recurrence risk of non-muscle invasive bladder cancer (NMIBC). Several randomized clinical trials (RCTs) have recently tested the efficacy of NBI-TUR for NMIBC.

**Objective:**

To perform a systematic review and meta-analysis of RCTs and evaluate the efficacy of NBI-TUR for NMIBC compared with white light imaging-assisted transurethral resection (WLI-TUR). The end point was recurrence risk.

Evidence acquisition: A systematic review of PubMed, Medline, Ovid, Embase, Cochrane and Web of Science was performed in February 2016 and updated in July 2016.

Evidence synthesis: Overall, six (*n* = 1084) of 278 trials were included. Three trials performed narrow band imaging-assisted electro-transurethral resection (NBI-ETUR), and two trials performed narrow band imaging-associated bipolar plasma vaporization (NBI-BPV). The last trial performed narrow band imaging-associated holmium laser resection (NBI-HLR). Statistical analysis was performed using Review Manager software (RevMan v.5.3; The Nordic Cochrane Center, Copenhagen, Denmark). The recurrence risk was compared by calculating risk ratios (RRs) with 95% confidence interval (CIs). Risk ratios with 95% CIs were calculated to compare 3-mo, 1-yr, and 2-yr survival rates. NBI-TUR was associated with improvements in the 3-mo recurrence risk (RR: 0.39; 95% CI, 0.26-0.60; *p* < 0.0001), 1-yr recurrence risk (RR: 0.52; 95% CI, 0.40-0.67; *p* < 0.00001) and 2-yr recurrence risk (RR: 0.60; 95% CI, 0.42-0.85; *p* = 0.004) compared with WLI-TUR.

**Conclusions:**

Compared with WLI-TUR, NBI-TUR can reduce the recurrence risk of NMIBC. The results of this review will facilitate the appropriate application of NBI in NMIBC.

## INTRODUCTION

Bladder cancer (BC) is a heterogeneous disease and the fourth most common malignant tumor, after prostate cancer, lung cancer, and colon cancer, in Western countries [1]. The incidence of BC is three to four times higher in men than in women. In the European Union, the incidence is 27 in 100000 for men and 6 in 100000 for women [2]. Fortunately, most newly diagnosed BCs are non-invasive urothelial tumors that are confined to the mucosa or mucosal lamina propria [3]. However, it is a long-term process from a predisposing change to relapse [4]. Although most poorly differentiated BCs do not progress, up to 20% of non-muscular infiltrating tumors can progress into myometrial invasion or metastasis [5]. It is a major challenge to reduce the high frequency of early recurrence risk of non-muscle invasive bladder cancer (NMIBC) because of the high recurrence rate, which can be as high as 45% at the first follow-up cystoscopy, 3 mo after TUR [6]. Bladder cancer recurrence and progression differ greatly with respect to tumor multiplicity, size, previous recurrence rates, T category, presence of carcinoma in situ (CIS) and grade [7]. In fact, most early “recurrences” are overlooked or residual tumors, and thus, it is important to increase BC visualization and detection for NMIBC. Neglected lesions will significantly affect the patient's management and outcome [8].

Micro-papillary or early flat in situ carcinomas can be difficult to detect by white light imaging (WLI) cystoscopy, but narrow band imaging (NBI) cystoscopy can improve the detection rate of recurrent flat and papillary superficial BC [9]. Herr and Donat evaluated recurrent BCs by WLI cystoscopy, followed by NBI cystoscopy, and found that 24% of patients had recurring cancer; 87% were detected by both WLI and NBI, and 100% were detected only by NBI cystoscopy. NBI cystoscopy can detect more papillary tumors or carcinoma in situ in 56% of recurrent patients [9]. NBI is an optical enhancement technology comprising two bandwidths of illumination centered on blue (415 nm) and green (540 nm). NBI can increase the contrast between the vasculature and superficial tissue structures of the mucosa [9]. In NBI mode, hemoglobin absorbs light through the tissue surface, thereby increasing the visibility of the capillary and surface structure. NBI improves tumor visibility by enhancing the contrast between vascularized lesions and normal mucosal [10–12]. These features allow NBI to detect small or flat early tumors that are difficult to observe by WLI [13]. Systems integrating both NBI and WLI are already available. Upon selection, a narrow-band filter is inserted in front of the white light source to activate the NBI mode [14, 15].

The majority of NMIBC recurrences after transurethral resection (TUR), particularly the intermediate and high levels of T1, are overlooked and residual cancers [16, 17]. The residual tumor rate after the first electro-TUR is approximately 33.8-36% [18, 19]. Among cases of NMIBC, 17.1% tumors were detected by NBI only, whereas 1.9% tumors were found by WLI only [20]. Additionally, 42% patients with positive urine cytology and negative WLI cystoscopy presented with BCs when undergoing NBI cystoscopy [21]. NBI cystoscopy facilitates the early detection and diagnosis of small lesions, reducing the rate of residual tumors.

Narrow band imaging-assisted transurethral resection (NBI-TUR) decreased the residual tumor rate significantly compared with white light imaging-assisted transurethral resection (WLI-TUR) in a matched cohort. The residual tumor rates at first follow-up for WLI-TUR and NBI-TUR were 30.5% and 15.0%, respectively [22]. Angelo found that NBI-UTR reduced the recurrence risk of NMIBC by at least 10% at 1 yr [23]. However, the study sample came from a single center and was limited, and we require many multi-center clinical trials for further verification. Thus, we aim to perform a systematic review and meta-analysis of randomized clinical trials (RCTs) that have evaluated the efficacy of NBI-TUR for NMIBC to provide the basis for clinical decision-making.

## EVIDENCE ACQUISITION

### Identification of eligible trials

The protocol was registered in the Prospective Register of Systematic Reviews (PROSPERO number CRD 42016038895). The study was designed and performed according to the Preferred Reported Items for Systematic Reviews and Meta-analysis guidelines [24]. Study designs considered for inclusion were random controlled trials and retrospective cohort studies assessing NBI-TUR for NMIBC.

The inclusion criteria were as follows: Participants: patients with NMIBC. Interventions: WLI-TUR and NBI-TUR as treatment modalities. Comparisons: WLI-TUR vs. NBI-TUR. Outcomes: recurrence risk.

The exclusion criteria were as follows: muscle invasive BC, non-comparative studies, no comparison between WLI-TUR and NBI-TUR, no detailed data regarding the recurrence risk in the WLI-TUR or NBI-TUR group.

The search was performed using PubMed, Medline, Ovid, Embase, Cochrane Library and Web of Science. We used the following search terms in all databases: (“narrow band imaging” or “NBI”) AND (“bladder cancer” or “bladder tumor” or “bladder carcinoma”) AND (“transurethral resection”). The references in the included articles were further examined to identify additional qualified clinical trials. Trials enrolling patients with NBI-TUR or WLI-TUR were included. When the same trial was reported more than once, the most recent information was considered in the analysis.

### Data collection

The following data were collected from each eligible trial, if available:

Main information: age, sex, clinical status, number of tumors, tumor grade, tumor stage, and tumor size.

Details of study treatment: modality of cystoscopy before TUR, type of TUR, modality of cystoscopy in TUR, adjuvant topical therapy after TUR.

Study design: primary end point, second end point, study type.

Patient enrollment and follow-up: start and end dates of the study; number of patients assigned to the experimental arm, number of patients assigned to the control arm, follow-up.

Recurrence risk: total number of patients, number of recurrences in each arm, p value.

The randomization quality was evaluated in each study based on the information available in the publication [23, 25–29].

### Statistical methods

After data extraction, the data were analyzed using Review Manager software (RevMan v.5.3; The Nordic Cochrane Center, Copenhagen, Denmark). Risk ratios (RRs) were used for dichotomous variables, with 95% confidence intervals (CIs). We evaluated the methodological heterogeneity among the selected literature, and we used the χ2 test and I2 scores to measure statistical heterogeneity. If the result was p>0.1 and I2 < 50%, we considered the heterogeneity low. We used a fixed-effects model to assess the data, and the p-value for significance was set at < 0.05.

## EVIDENCE SYNTHESIS

### Characteristics and quality of the trials

Supplementary Figure 1 shows the trial selection process for the search performed in February of 2016. Of 278 published papers, 272 were excluded, and 6 papers were eligible for inclusion [23, 25–29]. The patients with NMIBC in each trial were randomized into two arms using methods such as random permuted blocks and sealed envelopes.

The main analysis was performed considering the six comparisons of NBI-TUR vs. WLI-TUR. Three trials performed narrow band imaging-assisted electro-transurethral resection (NBI-ETUR) and WLI-assisted electro-TUR (WLI-ETUR) [23, 26, 27]. Two trials performed narrow band imaging-associated bipolar plasma vaporization (NBI-BPV) and WLI-ETUR [25, 29]. In those two trials, NMIBC was >3 cm in diameter in all patients. The last trial performed narrow band imaging-assisted holmium laser resection (NBI-HLR) and WLI-ETUR [28]. A holmium laser has high efficiency and safety features and provides a new method for the treatment of BC.

For the recurrence risk, a subgroup analysis was performed in patients according to the type of TUR, which included NBI-ETUR, NBI-BPV and NBI-HLR.

Figure 2 shows the quality assessment regarding the six available trials, as measured by the Cochrane Collaboration's tool for assessing the risk of bias. In all trials, patients assigned to the experimental arm received NBI-TUR, and the control arm received WLI-TUR. Five trials included in our analysis were RCTs, and one trial was a retrospective cohort that had a high risk of bias compared with the RCTs. One trial did not introduce the random sequence generation. All of the trials failed to blind the researchers to the treatment, which may affect the final results. Five trials exhibited a low risk of bias, and one study exhibited a high risk of bias.

**Figure 1:**
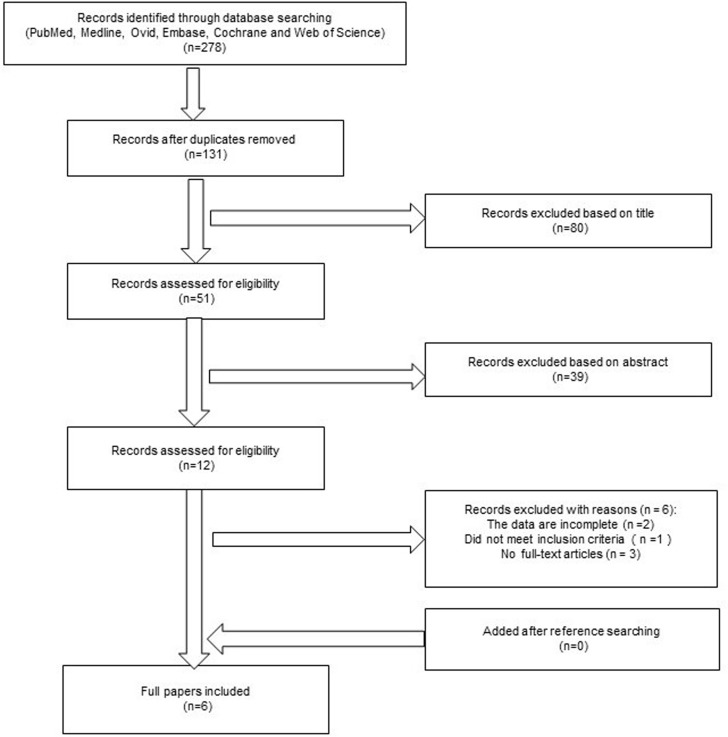
Preferred reporting items for the systemic review and meta-analysis flowchart Six papers were ultimately included.

**Figure 2:**
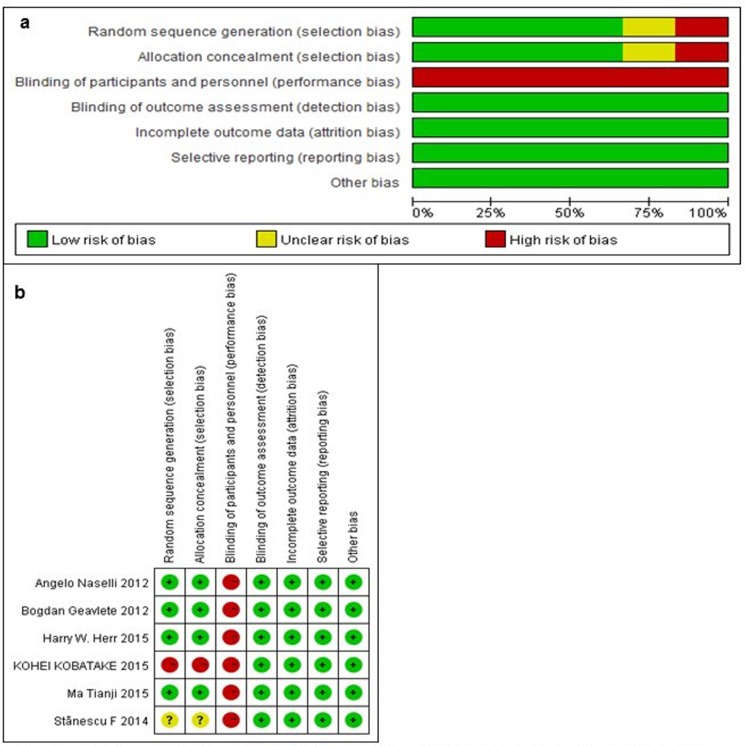
Risk of bias graph (a) and summary (b) Review author's judgments for each risk of bias item for each included study. Green: low risk of bias, red: high risk of bias and yellow: unclear risk of bias.

**Figure 3:**
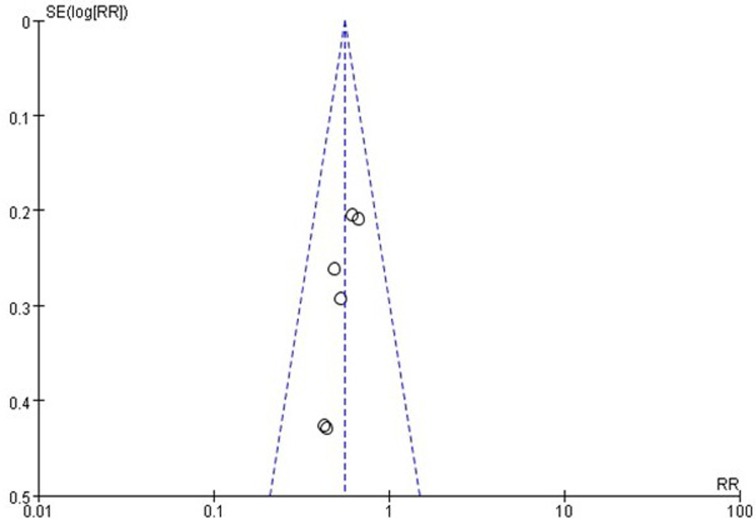
Funnel plot on recurrence risk Little evidence of publication bias was demonstrated of the funnel plots.

Regarding the BC recurrence risk, little evidence of publication bias was observed on visual or statistical examinations of the funnel plot (Figure 3).

### Patient characteristics

Overall, 1084 patients were included in the six trials in the meta-analysis; 556 (51.3%) were assigned to WLI-ETUR, and 528 (48.7%) were assigned to NBI-TUR, including 260 (24.0%) assigned to NBI-ETUR, 182 (16.8%) to NBI-BPV and 86 (8.0%) to NBI-HLR. The main characteristics of the 1084 patients are described in Table 1.

**Table 1:** Baseline characteristics of the included trials

Author/year	Angelo Naselli/2012				Harry W. Herr/2014				Kohei Kobatake/2015			Stănescu F/2014			Bogdan Geavlete/2011			Ma Tianji/2015		
	Total	WLI	NBI		Total	WLI	NBI		Total	WLI	NBI	Total	WLI	NBI	Total	WLI	NBI	Total	WLI	NBI
**Design**	A prospective randomized trial				A prospective randomized trial				A retrospective study			A prospective study			A prospective randomized trial			A prospective randomized trial		
**Sex, n**	148	72	76		254	127	127		135	78	57				220	110	110	178	92	86
** Male**	29	17	12		183	95	88		110	62	48				161	82	79	148	79	69
** Female**	119	55	64		71	32	39		25	16	9				59	28	31	30	13	17
**Age, median (range)**					68(42-99) 7(36-93)					73	75	64.8 (33-86) 5.2 (32-87)			64.5(30-83) 3.7(31-84)					
** Mean±SD**	71.6±12.470.8±10.3																		62±8	63±9
**Clinical status**									135	78	57									
** Recurrent**	65	28	37						78	45	33									
** Newly diagnosed**	83	44	39		254	127	127		57	33	24	ALL			ALL					
**No. of tumors, n**					254	127	127		135	78	57				220	110	110	178	92	86
** Single**	76	39	37		34	15	19		59	35	24				73	38	35	104	54	50
** Multiple**	72	33	39		220	112	108		76	43	33	NA			147	72	75	74	38	36
**Grade**									135	78	57									
								1	11	8	3									
** Low**		41	39					2	87	51	36	NA				NA				
** High**		31	37		254	127	127	3	37	19	18									
**Stage**					254	127	127		135	78	57				179	90	89			
** T0**																				
** Ta**	110	52	58		161	81	80		106	61	45				50	26	24			
** Tis**									8	5	3									
** T1**	38	20	18		93	46	47		21	12	9				129	64	65			
**Tumor size**					254	127	127		135	78	57							178	92	86
				<1-2cm	19	9	10													
** ≤3 cm**	108	53	55	2-5cm	194	97	97		122	69	53							128	63	65
** ≥3 cm**	40	19	21	>5cm	41	21	20		13	9	4	ALL			ALL			50	29	21
**Adjuvant intravesical therapy**					6-wk induction				NA											
** No therapy**	91	49	42									All immediate instillation: doxorubicin or epirubicin; Re-TUR: BCG(1-yr)			ALL			All immediate instillation: Pirarubicin; The intermediate and high risk: Pirarubicin(1-yr)		
** BCG**	43	19	24		254	127	127													
** Mitomycin**	14	4	10																	
** Pirarubicin**																				
**follow-up**	11 mo (range 2–19 mo)				A minimum of 2 yr					1 yr										
**Exclusion criteria**	invasive BCa; without followup; absence pathologic				NA				Received post-operative intravesical injections of			muscle-invasive bladder cancer			muscle-invasive bladder cancer			Muscle-invasive bladder cancer, No received		
**Operative process**	WLI-ETUR: WLI cystoscopy—WLI-ETUR;				WEI-ETUR: WLI cystoscopy—WLI-ETUR; NBI-ETUR: WLI cystoscopy–NBI cystoscopy–NBI-ETUR-WLI and NBI cystoscopy				WLI-ETUR: WLI cystoscopy—WLI-ETUR; NBI-ETUR: WLI cystoscopy—NBI cystoscopy—NBI-ETUR			WIL-ETUR: WLI cystoscopy—WLI-ETUR; NBI-BPV: WLI cystoscopy–NBI cystoscopy–WLI and NBI-BPV			WIL-ETUR: WLI cystoscopy—WLI-ETUR; NBI-BPV: WLI cystoscopy–NBI cystoscopy–WLI and NBI-BPV			WLI-ETUR: WLI cystoscopy—WIL-ETUR; NBI cystoscopy–WLI and NBI-HLR		

### Recurrence risk

**Figure 4:**
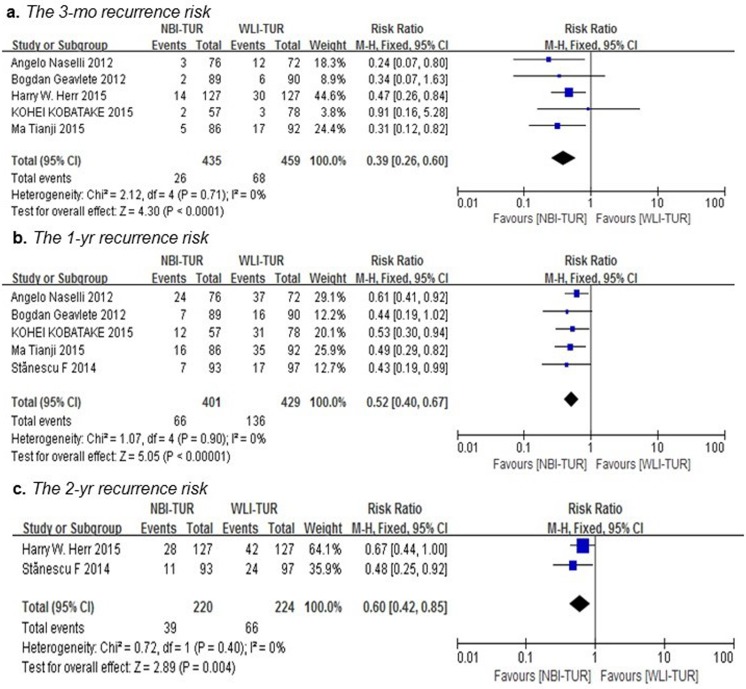
Pairwise meta-analysis for the recurrence risk of NMIBC during the follow-up period NBI-TUR was associated with a significant benefit in the 3-mo recurrence risk **a**., 1-yr recurrence risk **b**. and 2-yr recurrence risk **c**.

Overall, cancer recurred in 283 patients in the two arms during the follow-up period, as shown in Table 2. As shown in Figure 4, NBI-TUR in NMIBC was associated with a significant benefit in the 3-mo (RR: 0.39; 95% CI, 0.26-0.60; p < 0.0001), 1-yr (RR: 0.52; 95% CI, 0.40-0.67; p < 0.00001) and 2-yr (RR: 0.60; 95% CI, 0.42-0.85; p = 0.004) recurrence risks compared with WLI-TUR. There was no evidence of significant heterogeneity among the trials (p = 0.71; I2 = 0%), (p = 0.90; I2 = 0%) and (p = 0.40; I2 = 0%), respectively.

**Table 2:** The recurrence risk of NMIBC during the follow-up period

		WLI					NBI				
Author/year	Patients, n	Operation method	3-mo RR, n	1-yr RR, n	2-yr RR, n	Total patients, n	Operation method	3-mo RR, n	1-yr RR, n	2-yr RR, n	Total patients, n
Angelo Naselli/2012	148	ETUR	12	37		72	ETUR	3	24		76
Harry W. Herr/2014	254	ETUR	30		42	127	ETUR	14		28	127
Kohei Kobatake/2015	135	ETUR	3	31		78	ETUR	2	12		57
Stănescu F/2014	260	ETUR		17	24	97	BPV		7	11	93
Bogdan Geavlete/2011	220	ETUR	6	16		90	BPV	2	7		89
Ma Tianji/2015	209	ETUR	17	35		92	HLR	5	16		86
Total	1226	TUR	Total RR,n: 185			556	TUR	Total RR,n: 98			528

**Figure 5:**
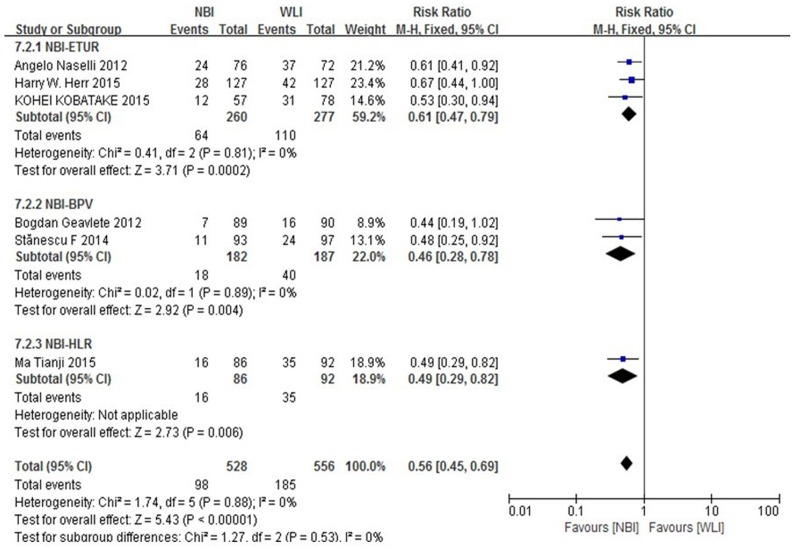
Subgroup analysis for operation method Subgroup analyses did not show that different surgical methods for recurrence of NMIBC had a significant interaction.

A subgroup analysis was performed for all patients with operation methods of the experimental group enrolled in the six trials (Figure 5). Subgroup analyses did not show that different surgical methods for recurrence of NMIBC had a significant interaction (p = 0.53). The RR for the operation method was 0.61 (95% CI, 0.47-0.79) in patients with NBI-ETUR, 0.46 (95% CI, 0.28-0.78) in patients with NBI-BPV and 0.49 (95% CI, 0.29-0.82) in patients with NBI-HLR.

## DISCUSSION

This meta-analysis shows that NBI-TUR for NMIBC has a significant improvement in recurrence risk compared with WLI-TUR.

A quantitative synthesis of the currently available evidence regarding this operation method could be very helpful for clinical decisions because these six trials showed a significant benefit associated with NBI-TUR for the treatment of NMIBC. To the best of our knowledge, no other trials have reported the recurrence risk of NBI-TUR in NMIBC, and this meta-analysis synthesizes all the evidence produced to date. Although all the trials showed the benefit of NBI-TUR for NMIBC in terms of the recurrence risk, the study sample was a single-center study and was limited, and we require many multi-center clinical trials to further verify the findings. Therefore, this meta-analysis synthesizes six trials to test the efficacy of NBI-TUR in NMIBC.

Approximately 70% of cases of NMIBC present with pTa, 20% with pT1, and 10% with CIS lesions [1]. Many studies have reported routine detection of residual tumors during second TUR [30]. For example, Babjuk et al. [31] reported tumors in 32-36% of patients at a second TUR within 7 weeks after the initial TUR, particularly in patients with TaG3 disease or multiple tumors [31, 32]. Residual transitional cell tumors were observed in 44% of patients after re-resection of the original tumor site [16]. For high-grade tumors, this rate could be as high as 70% [33]. The probabilities of recurrence after TUR at 1 yr ranged from 15% to 61%, and the probabilities of recurrence ranged from 31% to 78% at 5 yr [7]. Sylvester et al. reported that the median time to first recurrence was 2.7 yr [7]. The high rate of recurrence is a major challenge when treating NMIBC. Brausi et al. showed that overlooked tumors during the initial TUR, not true relapse, are responsible for recurrence. The rate of ‘recurrences’ was 3.4-20.6% for single tumors and 7.4-45.6% for multiple tumors [6]. Therefore, some researchers have suggested that recurrence can be divided into true recurrence and residual tumors that were likely overlooked on the initial TUR. Improving the detection rate of NMIBC will help reduce recurrences.

To date, the standard operation is WLI-TUR, but this operation might miss small papillary tumors or CIS. The detection rate of NBI was 94.7% vs. 79.2% for WLI (p < .001). NBI cystoscopy as optical enhancement technology improves the detection of primary and recurrent NMIBC over WLI [34]. Although some trials have proven the advantage of using NBI-TUR, for example Herr [26] reported an 11% reduction in the recurrence rate using NBI-TUR and Naselli et al. [23] showed that NBI-TUR reduces the 1-yr recurrence risk of NMIBC by at least 10%, these trials were single-center studies and were limited [23, 26].

Our systematic review could be regarded as a multi-center, large sample study, but it also has some limitations. First, not all experiments were prospective randomized controlled trials. The study by Kobatake et al. [27] was a retrospective cohort study. Major biases with retrospective cohort studies could impact the recall of former exposure to risk variables, including selection bias and information bias. It could be very difficult to make accurate comparisons between the NBI-TUR and WLI-TUR arms. Second, in five trials, the operative process was unreasonable, and NBI cystoscopy was regarded as a second procedure to inspect and resect visible or suspected tumors. This process would result in observer bias because NBI would be used as a supplement to conventional WLI and may prove to be better than either modality used alone. Furthermore, operators may break blindness and take more time in NBI-TUR than in WLI-TUR. Third, the design of the experimental group was not perfect. In the control group, all patients with NMIBC in six trials underwent WLI-ETUR, whereas in the experimental group, patients with NMIBC in three trials underwent NBI-ETUR, patients in two trials underwent NBI-BPV and patients in one trial underwent NBI-HLR. The different operation methods between the experimental group and control group would affect the recurrence risk of NMIBC. Fourth, cost and time implication analyses for NMIBC in the NBI-TUR were not performed. Fifth, intensive analyses of different sub-groups based on the clinical characteristics of patients are needed to compare recurrence rates. Gender, age, tumor stage, grade and adjuvant topical therapy should also be considered in future analyses. These factors are associated with the recurrence risk, but the lack of complete data limited our ability to analyze these factors. Finally, long-term recurrence risk and overall survival analyses are needed.

Despite the above limitations, we still believe our review has clinical significance. All trials showed that NBI-TUR reduced the recurrence risk of NMIBC compared with WLI-TUR, in particular Ma et al. reported the 1-yr recurrence risk was 18.60% in the NBI-HLR and 38.04% in the WLI-ETUR [28]. NBI-HLR reduced the 1-yr recurrence risk significantly by 19.44%. Geavlete et al. reported a slight difference of 9.9% in the 1-yr recurrence risk for NMIBC>3 cm, with rates of 7.9% for NBI-BPV vs. 17.8% for WLI-ETUR [25]; however, the recurrence risk was higher than that reported by Ma et al. [28]. In another study, the 1-yr recurrence risk after NBI-BPV compared with WLI-ETUR was 7.2% vs. 18.3%, respectively. That study also reported that the 2-yr recurrence risk after NBI-BPV was 11.5% vs. 25.8% for WLI-ETUR [29]. Herr reported that the 2-yr recurrence risk was 22% vs. 33%, representing a reduction of 11% for NBI-TUR, and the 2-yr recurrence-free survival and mean survival time were 22 mo and 19 mo, respectively [26]. Kobatake et al. reported that the recurrence probability increased in the NBI group from 3.5% at 3 mo to 21.1% at 1 yr and increased from 3.8% to 39.7% in the control group [27]. The last trials showed that the recurrence rates increased from 4% at 3 mo to 32% at 1 yr for NBI-TUR and from 12% to 51% for WLI-TUR. Because of the increasing trend of tumor recurrence, some recurrence is attributed to causes other than missed tumors, such as the field cancerization effect [35] or the clonal origin of urothelial cancer [13], which are not yet completely understood. The recurrence mechanism of NMIBC should be further studied.

One aspect to consider is whether we are ready to change practices based on this review. First, the above trials showed that NBI-TUR can reduce the recurrence risk of NMIBC compared with WLI-TUR at 3 mo, 1 yr and 2 yr. Photodynamic diagnosis (PDD) is another new imaging technology that has been recently introduced. PDD requires the perfusion of photoactive porphyrin precursors, such as 5-aminolevulinic acid (5-ALA) or hexylaminolevulinate (HAL), and could improve the detection rate of BC. Fluorescence cystoscopy improves the detection rate of papillary tumors [36] and CIS [37] compared with WLI cystoscopy. In a randomized prospective study of treatment outcomes, the tumor-free recurrence rate at 1-yr after TUR using 5-ALA was 18% lower than that after WLI-TUR [38]. A review reported that the recurrence rate among resected patients was not significantly different between either 5-ALA or HAL and NBI-TUR [39]. Second, recurrence increases the workload and the diagnosis, treatment and, in particular, follow-up of NMBIC, thus increasing costs [32]. Botteman et al. [40] calculated that BC is currently the most expensive tumor because existing diagnosis, treatment and follow-up methods are not cost-effective. Avritscher et al. [41] calculated comparable costs and found that the treatment of recurrences accounts for approximately 60% of these costs. Third, PDD requires perfusion of photosensitizers, whereas NBI cystoscopy does not require additional invasive procedures [42]. NBI-TUR is superior to PDD because the specificity of PDD for the diagnosis of BC is significantly decreased in the patients who were treated with bladder instillation [43]. In addition to the drawback of photodynamic diagnostics, the relatively low specificity of PDD must be considered [44]. We believe that NBI-TUR should be further studied and generalized.

However, NBI cystoscopy has limitations. First, because NBI has no tumor specificity and can only provide morphological features of suspicious lesions, this increased sensitivity and decreased specificity will eventually lead to an increased rate of false-positives [45]. Second, bladder instillation to detect lesion recurrence due to the lack of NBI examination standards may lead to unnecessary biopsy [46]. Third, the light emitted from abnormal lesions is strongly absorbed by hemoglobin, and thus, abnormal conditions such as bladder bleeding or active inflammation make NBI difficult to diagnose [47].

This review will aid the more precise identification of indications for the application of NBI in NMIBC.

## CONCLUSIONS

Our meta-analysis clearly shows NBI-TUR can significantly reduce the recurrence risk of NMIBC compared with WLI-TUR at 3 mo, 1 yr and 2 yr. Considering the absence of heterogeneity among the six trials, the result is believable. If our findings are confirmed by large multi-center validating studies, NBI-TUR would be used in the clinic.

## Abbreviations

WLI: white light imaging
NBI: narrow band imaging
TUR: transurethral resection
BC: bladder cancer
NMIBC: non-muscle invasive bladder cancer
NBI-TUR: narrow band imaging-assisted transurethral resection
WLI-TUR: white light imaging-assisted transurethral resection
WLI-ETUR: white light imaging-assisted electro-transurethral resection
NBI-ETUR: narrow band imaging-assisted electro-transurethral resection
NBI-BPV: narrow band imaging-associated bipolar plasma vaporization
NBI-HLR: narrow band imaging-assisted holmium laser resection
RCT: randomized clinical trial
CI: confidence interval
RR: risk ratio
mo: month
yr: year
CIS: carcinoma in situ
PDD: photodynamic diagnosis
5-ALA: 5-aminolevulinic acid
HAL: hexylaminolevulinate
